# Occurrence and Severity of Catheter-Related Bladder Discomfort of General Anesthesia Plus Epidural Anesthesia vs. General Anesthesia in Abdominal Operation With Urinary Catheterization: A Randomized, Controlled Study

**DOI:** 10.3389/fsurg.2021.658598

**Published:** 2021-09-06

**Authors:** Shunxiang Sun, Cheng Wang, Jun Zhang, Pengfei Sun

**Affiliations:** ^1^Department of Critical Care Medicine, Ruijin Hospital, Shanghai Jiaotong University School of Medicine, Shanghai, China; ^2^Department of Anesthesiology, Fudan University Shanghai Cancer Center, Shanghai, China

**Keywords:** epidural anesthesia, general anesthesia, catheter-related bladder discomfort, urinary catheterization, adverse events

## Abstract

**Background:** This randomized, controlled study aimed to investigate the effect of general anesthesia plus epidural anesthesia on catheter-related bladder discomfort (CRBD) in patients who underwent abdominal operation with urinary catheterization.

**Methods:** A total of 150 patients scheduled for abdominal operation under anesthesia with urinary catheterization were randomized to receive general anesthesia plus epidural anesthesia (*N* = 74, GA + EA group) or general anesthesia (*N* = 76, GA group). The occurrence and severity of CRBD, systolic blood pressure (SBP), diastolic blood pressure (DBP), and heart rate (HR) were recorded at 0 hour (h), 0.5, 1, and 3 h after tracheal extubation. Besides, postoperative adverse events were assessed.

**Results:** The occurrence and severity of CRBD at 0, 0.5, 1, and 3 h were all reduced in GA + EA group compared to GA group (all *P* < 0.05). Meanwhile, subgroup analyses showed that the reduction of occurrence and severity of CRBD in GA + EA group compared to GA group was more obvious in male patients and patients ≥50 years. Besides, SBP at 0, 0.5, 1, and 3 h, as well as DBP at 0, 0.5, and 3 h were all decreased in GA + EA group compared to GA group (all *P* < 0.05), while HR was increased at 0 h in GA + EA group compared to GA group (*P* = 0.034). Moreover, the occurrence of pain, severity of pain and occurrence of vomiting were similar between GA + EA group and GA group (all *P* > 0.05).

**Conclusion:** General anesthesia plus epidural anesthesia decreases CRBD occurrence and severity with tolerable safety compared with general anesthesia in patients who undergo abdominal operation with urinary catheterization.

## Introduction

Urinary catheterization is vastly performed during various abdominal operations, while it might lead to several adverse events including urinary tract infection and catheter-related bladder discomfort (CRBD) (1, 2). CRBD, of which incidence ranges from 20 to 90%, increases patient's stresses both physiologically and psychologically, and prolongs the length of recovery as well as hospital stay (3). Currently, muscarinic receptor antagonists (such as oxybutynin and tolterodine), anesthetics and analgesics (such as ketamine and tramadol) are administrated for the prevention and treatment of CRBD; however, considering the occurrence of CRBD is still high under the administration of these agents (4–6), searching for novel strategies for the treatment and prevention of CRBD is critical.

General anesthesia is one of the most common options for patients undergoing operation, and its combination with locoregional anesthesia is popular in recent decades, among which general anesthesia plus epidural anesthesia is widely performed in various operations (7, 8). Epidural anesthesia is proved to provide a good anesthetic effect with acceptable tolerance (9). Besides, it possesses potential regulation on urinary tract function: previous studies suggest that epidural anesthesia reduces bladder sensitivity and decreases the urge to void possibly by inhibiting detrusor activity and bladder contraction, and the latter ones are partly regulated by the sympathetic fiber in the intermediolateral cell column of the spinal cord (T11-L2) (10–13). Since the CRBD is mechanically similar to the overreactive bladder caused by bladder involuntary contraction (14), we hypothesized the general anesthesia plus epidural anesthesia might reduce the occurrence of CRBD with good tolerance compared to general anesthesia in patients who underwent abdominal operation with urinary catheterization. However, the relevant information is lacking.

This randomized, controlled study aimed to compare the effect of general anesthesia plus epidural anesthesia vs. general anesthesia on the occurrence and severity of CRBD in patients who underwent abdominal operation with urinary catheterization.

## Methods

### Patients

This randomized, controlled study was conducted between January 2020 and May 2020. Totally, 150 patients scheduled for abdominal operation under anesthesia with urinary catheterization were recruited in this study. The inclusion criteria were: (1) scheduled for abdominal operation; (2) requiring urinary catheterization; (3) age ≥18 years; (4) American Society of Anesthesiologists (ASA) grade I–II; (5) able to communicate properly. The exclusion criteria included: (1) coagulation disorders or cutaneous disorders precluding safe epidural catheterization; (2) overactive bladder which was defined as frequency >3 times in the night or >8 times in 24 h; (3) known neurogenic bladder or bladder outflow obstruction; (4) long-term use of chronic analgesics; (5) history of urinary catheterization; (6) pregnant or lactating women. This study was approved by the Institutional Review Board, and all participants signed the informed consents.

### Random Allocation

Before the operation, eligible patients were randomly allocated to the GA group (*N* = 76) or GA + EA (*N* = 74) group. The random allocation sequence was generated using the block randomization method with a block size of 4 by an analyst who was not involved in the patients' assignment. One random allocation code was corresponded to one patient ID and sealed in an opaque envelope. When a patient's eligibility was confirmed, a unique patient ID was generated, then the corresponding envelope was opened, subsequently, the allocation of the patient was determined.

### Anesthesia Procedures

(1) GA group: anesthesia was induced with sufentanil (Yichang Humanwell Pharmaceutical Co., Ltd, China) 0.2–0.5 μg/kg, propofol (AstraZeneca, UK) 1.5–2.5 mg/kg, rocuronium (Organon, Netherlands) 0.6 mg/kg, and continuously-pumped remifentanil (GlaxoSmithKline, UK) at 2–4 ng/mL. Before surgical incision, 10 μg sufentanil was administered, which was then added during operation at a dose of 5–10 μg every hour. Anesthesia was maintained by general anesthesia with sevoflurane (Abbott, USA) plus continuous pumping of 2–4 ng/mL remifentanil. Near the end of the operation, 3 mg granisetron (GlaxoSmithKline, UK) was administered. After the operation, neostigmine (Shanghai Xinyi Jinzhu Pharmaceutical Co., Ltd., China) and atropine (Shanghai Xianding Pharmaceutical Co., Ltd., China) were used to antagonize muscle relaxation. Analgesic pump comprised of follows: sufentanil 100 μg, flurbiprofen (Beijing Tide Pharmaceutical Co., Ltd., China) injection 200 mg, granisetron 3 mg, and normal saline 200 mL, and it had a total amount of 220 mL, continuous and bolus dose of 3.5 mL. After the operation, patients were transferred to the post-anesthesia care unit (PACU). When patients were resuscitated in the PACU, the tracheal extubation was administered. Finally, patients were sent to the inpatient ward after they had stable vital signs.

(2) GA + EA group: the epidural catheter was inserted in T10-T11, T11-T12, or T12-L1 of the thoracolumbar junction, as appropriate (15–17). When the epidural intubation was completed, lidocaine (Shanghai Zhaohui Pharmaceutical Co., Ltd., China) (1%, 3 mL) was used as a test dose, and the anesthesia induction was administered with sufentanil 0.2–0.5 g/kg, propofol 1.5–2.5 mg/kg, rocuronium 0.6 mg/kg, and continuously-pumped remifentanil at 2–4 ng/mL. Before surgical incision, 10 μg sufentanil and 4 mL 0.25% ropivacaine (AstraZeneca, UK) were administered, then ropivacaine was added in epidural during operation at a dose of 4 mL (0.25%) every hour. Anesthesia was maintained by epidural anesthesia and inhaled general anesthesia with sevoflurane plus continuous pumping of 2–4 ng/mL remifentanil. Near the end of the operation, 3 mg granisetron was administered. After the operation, neostigmine and atropine were used to antagonize muscle relaxation. Analgesic pump comprised of follows: ropivacaine 300 mg, sufentanil 100 μg, and normal saline 200 mL, which had a total amount of 230 mL, continuous and bolus dose of 3.5 mL. After the operation, patients were transferred to the post-anesthesia care unit (PACU). When patients were resuscitated in the PACU, the tracheal extubation was administered. Finally, patients were sent to the inpatient ward after they had stable vital signs.

After anesthesia induction, urinary catheterization was performed in both two groups by nurses using a 16F Foley catheter (its balloon inflated with 10 mL of normal saline). The catheter was lubricated in advance, fixed to the pubic arch by adhesive tape to avoid pulling, and connected to the catheterization bag. Catheter indwelling time ranged from 24 to 48 h.

### Assessment

The CRBD was characterized by an urge to pass urine or discomfort in the suprapubic region, which was assessed at 0, 0.5, 1, and 3 h after tracheal extubation in the PACU, using a 4-point scale (3): none (0 points), had no complaint of any CRBD even on being questioned; mild (1 point), reported by the patient only on being questioned; moderate (2 points), reported by the patient initiatively without questioning but not accompanied by any behavioral responses; severe (3 points), reported by the patient without questioning and accompanied by behavioral responses. Behavioral responses were referred to flailing limbs, strong vocal response and attempts to pull out the urinary catheter. Besides, systolic blood pressure (SBP), diastolic blood pressure (DBP), and heart rate (HR) were measured at 0, 0.5, 1, and 3 h after tracheal extubation in the PACU, and postoperative adverse events were recorded as well. Pain was assessed by Numerical Rating Scale (18), by which point 0 was categorized as “none,” point 1–3 was categorized as “Mild,” point 4–6 was categorized as “Moderate” and point 7–10 was categorized as “Severe.”

### Sample Size Estimation

The incidence of CRBD reported in a previous study was 55% (3). We hypothesized that GA + EA could reduce the incidence of CRBD to 30%. That was, the CRBD proportion in the GA + EA group was assumed to be 0.30, and the proportion in the GA group was 0.55. The test statistic used was the two-sided *Z* test with pooled variance. A sample size of 120 in total achieved 81% power to detect a difference between the group proportions of −25%. Considering the possible dropouts in each group, we increased the sample size to 150 in total, which could achieve a power of 0.88.

### Statistical Analysis

Variables were described as the number with percentage or mean with standard deviation (SD). Characteristics comparison between two groups was analyzed by Student's *t*-test or Chi-square test. Comparison of incidence and severity of CRBD between two groups was analyzed by Chi-square test and Wilcoxon rank-sum test, respectively. Comparison of SDP, BDP and HR at different assessed time points was analyzed by Student's *t*-test. Comparison of adverse events was analyzed by Chi-square test, and Comparison of pain severity was analyzed by Wilcoxon rank-sum test. SPSS 22.0 (IBM, Chicago, Illinois, USA) and GraphPad Prism 7.01 (GraphPad Software Inc., San Diego, California, USA) were applied for statistical analysis and diagram making. *P*-value < 0.05 was considered statistically significant.

## Results

### Study Flow

**Figure 1:**
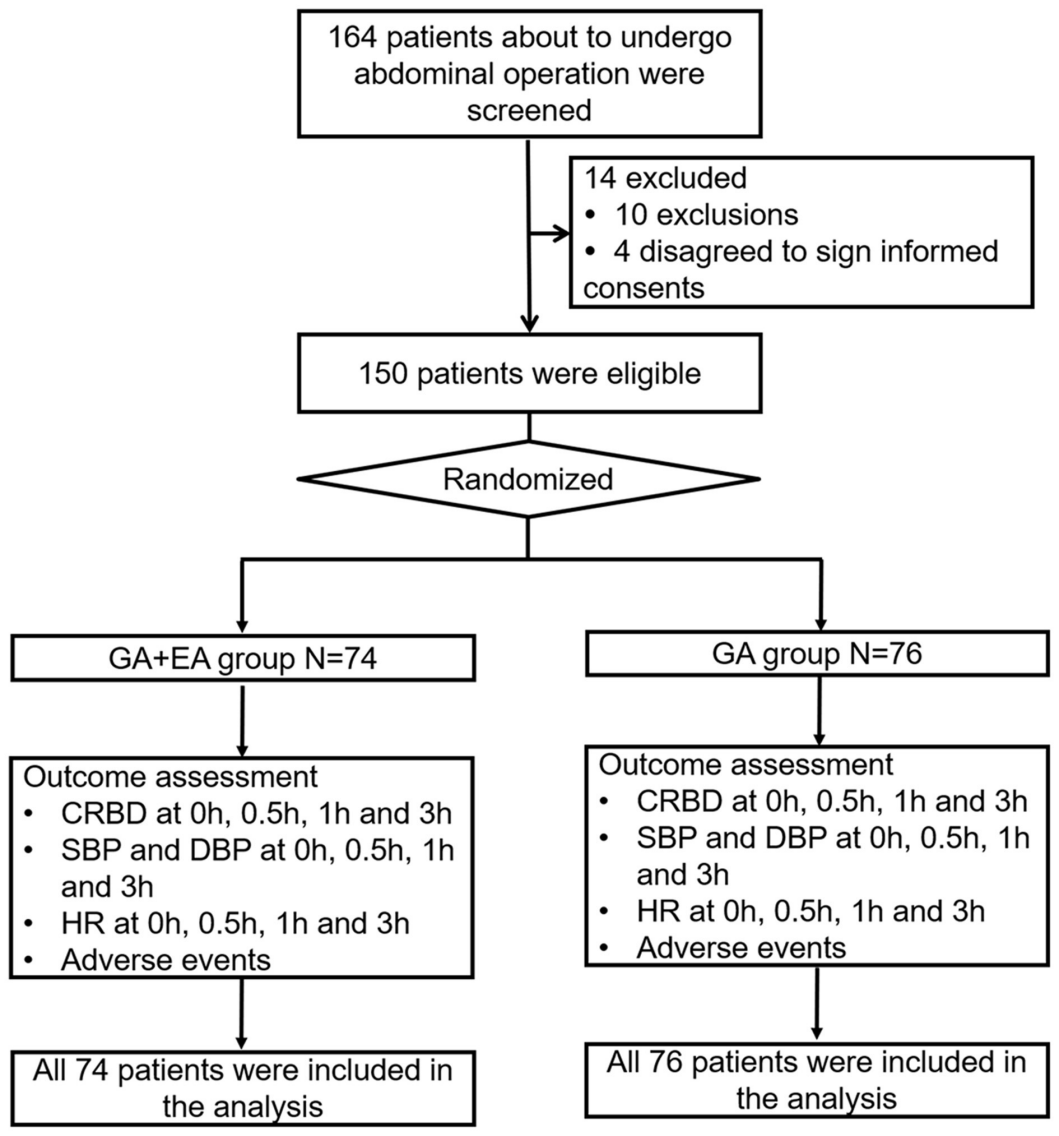
Flow chart. GA, general anesthesia; EA, epidural anesthesia; CRBD, catheter-related bladder discomfort; SBP, systolic blood pressure; DBP, diastolic blood pressure; HR, heart rate.

A total of 164 patients about to undergo abdominal operation were screened for eligibility, among which 14 patients were excluded (including 10 patients who either did not meet the inclusion criteria or met the exclusion criteria, and four patients who disagreed to sign informed consents). Subsequently, 150 eligible patients were randomized into the GA + EA group (*N* = 74) and GA group (*N* = 76). In both groups, outcomes were assessed, which included CRBD, SBP, DBP and HR at 0, 0.5, 1, and 3 h, as well as adverse events. Finally, all eligible patients were included in the analyses (Figure 1).

### Comparison of Basic Characteristics Between Groups

The GA + EA group had a mean age of 50.1 ± 9.8 years with 32 (43.2%) males and 42 (56.8%) females; meanwhile, the GA group had a mean age of 51.9 ± 9.7 years with 24 (31.6%) males and 52 (68.4%) females. The comparison analyses showed that no difference was found in age, gender distribution, weight, ASA grade or type of surgery between the two groups (all *P* > 0.05) (Table 1). In addition, the specific operations and operative regions or organs of patients were shown in Supplementary Tables 1, 2, respectively.

**Table 1:** Characteristics of patients.

**Parameters**	**GA + EA (*N* = 74)**	**GA (*N* = 76)**	***P*-value**
Age (years), mean ± SD	50.1 ± 9.8	51.9 ± 9.7	0.274
Gender, No. (%)			0.140
Male	32 (43.2)	24 (31.6)	
Female	42 (56.8)	52 (68.4)	
Weight (kg), mean ± SD	63.2 ± 14.1	60.5 ± 10.6	0.185
ASA grade, No. (%)			0.776
I	16 (21.6)	15 (19.7)	
II	58 (78.4)	61 (80.3)	
Type of surgery, No. (%)			0.354
Digestion system-related surgery	26 (35.2)	28 (36.8)	
Urinary system-related surgery	8 (10.8)	13 (17.1)	
Gynecology-related surgery	28 (37.8)	29 (38.2)	
Others	12 (16.2)	6 (7.9)	

*GA, general anesthesia; EA, epidural anesthesia; SD, standard deviation; ASA, american society of anesthesiologists*.

### Comparison of CRBD Between Groups

**Figure 2:**
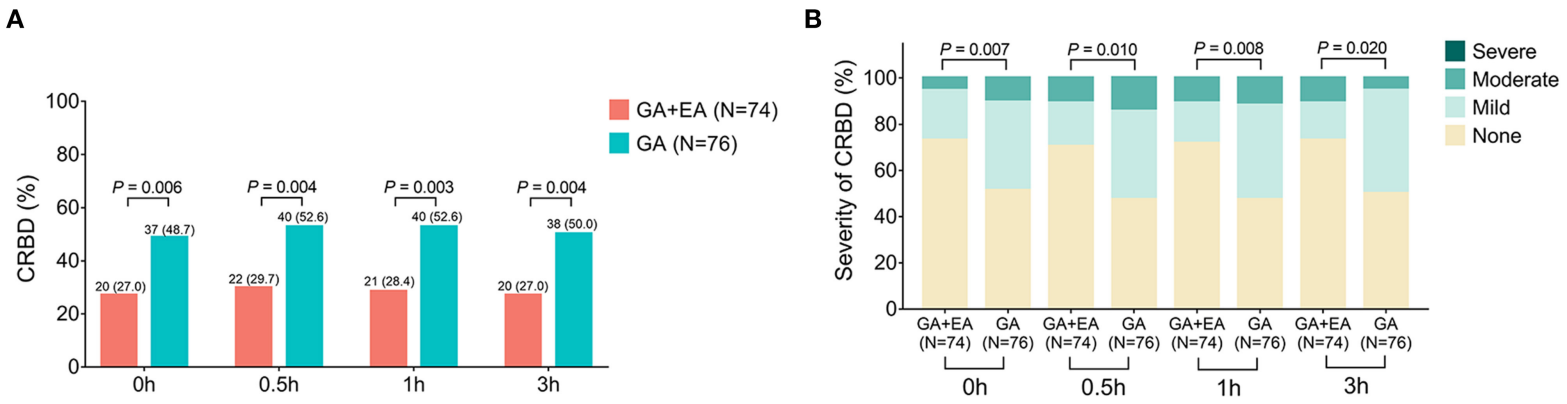
Occurrence and severity of CRBD. Comparison of the occurrence **(A)** and severity **(B)** of CRBD between GA + EA group and GA group. CRBD, catheter-related bladder discomfort; GA, general anesthesia; EA, epidural anesthesia; h, hour.

**Figure 3:**
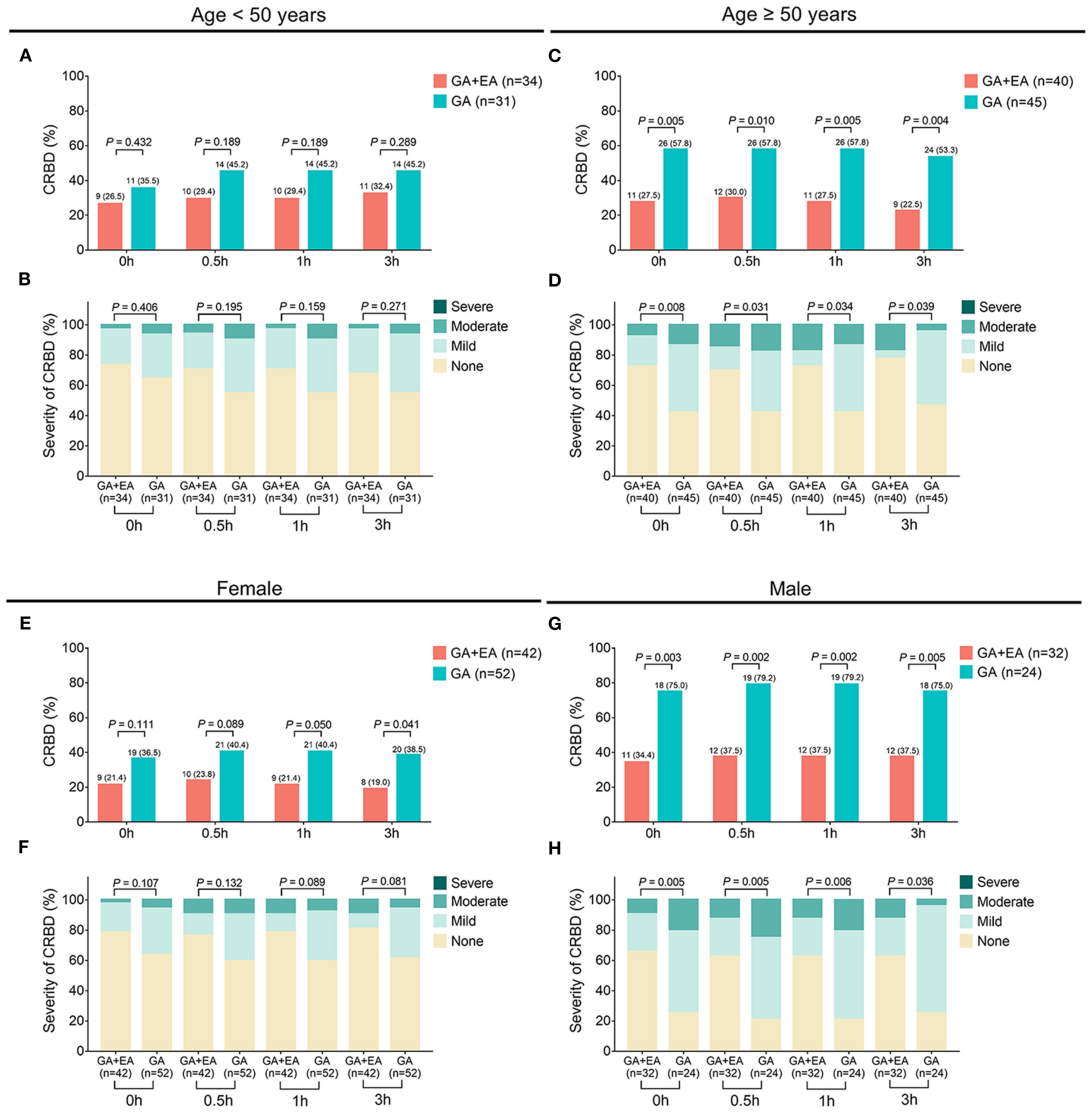
Occurrence and severity of CRBD in subgroups. Comparison of the occurrence **(A)** and severity **(B)** of CRBD between GA + EA group and GA group in patients younger than 50 years; Comparison of the occurrence **(C)** and severity **(D)** of CRBD between GA + EA group and GA group in patients equal to or older than 50 years; Comparison of the occurrence **(E)** and severity **(F)** of CRBD between GA + EA group and GA group in female patients; Comparison of the occurrence **(G)** and severity **(H)** of CRBD between GA + EA group and GA group in male patients. CRBD, catheter-related bladder discomfort; GA, general anesthesia; EA, epidural anesthesia; h, hour.

**Figure 4:**
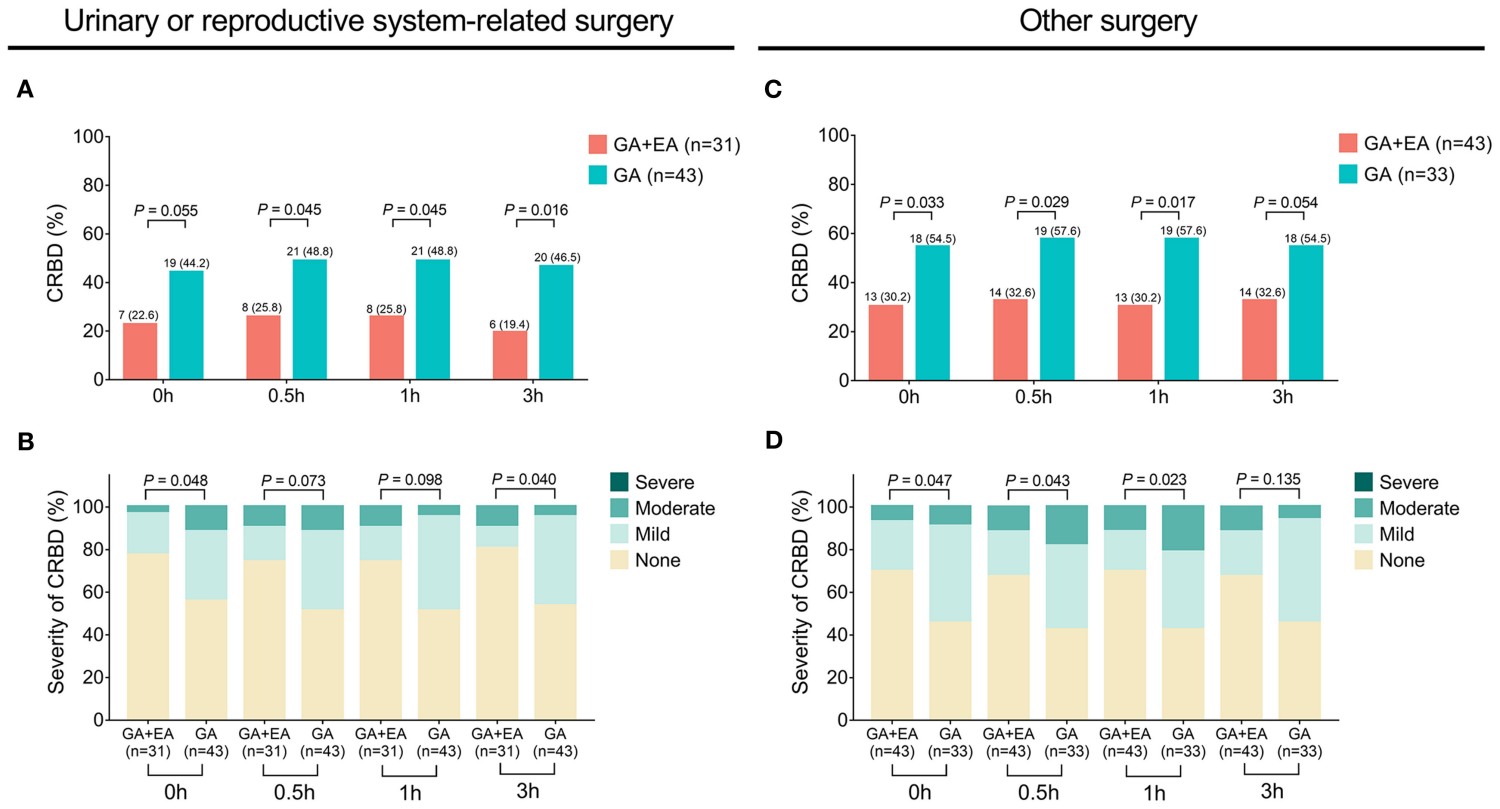
Occurrence and severity of CRBD in patients with urinary/reproductive system-related surgeries or other surgeries. Comparison of the occurrence **(A)** and severity **(B)** of CRBD between GA + EA group and GA group in patients with urinary or reproductive system-related surgeries; Comparison of the occurrence **(C)** and severity **(D)** of CRBD between GA+EA group and GA group in patients with other surgeries. CRBD, catheter-related bladder discomfort; GA, general anesthesia; EA, epidural anesthesia; h, hour.

The occurrence of CRBD at 0 h (*P* = 0.006), 0.5 h (*P* = 0.004), 1 h (*P* = 0.003) and 3 h (*P* = 0.004) was all reduced in the GA + EA group compared with the GA group (Figure 2A). Meanwhile, the severity of CRBD at 0 h (*P* = 0.007), 0.5 h (*P* = 0.010), 1 h (*P* = 0.008) and 3 h (*P* = 0.020) was also decreased in the GA + EA group compared with the GA group (Figure 2B). Further subgroup analyses revealed that in patients equal to or older than 50 years (Figures 3C,D) and male patients (Figures 3G,H), the occurrence and severity of CRBD at 0, 0.5, 1, and 3 h were reduced in GA + EA group compared with GA group. However, in patients younger than 50 years (Figures 3A,B) and female patients (Figures 3E,F), the occurrence and severity of CRBD at 0, 0.5, 1, and 3 h almost did not vary obviously between the two groups. Moreover, in patients with urinary or reproductive system-related surgery, the occurrence of CRBD at 0.5, 1, and 3 h and severity of CRBD at 0 and 3 h were reduced in GA+EA group compared with GA group; meanwhile, the occurrence and severity of CRBD at 0, 0.5 and 1 h were also decreased in GA + EA group compared with EA group in patients with other surgeries (all *P* < 0.05) (Figures 4A–D).

### Comparison of SBP and DBP Between Groups

**Figure 5:**
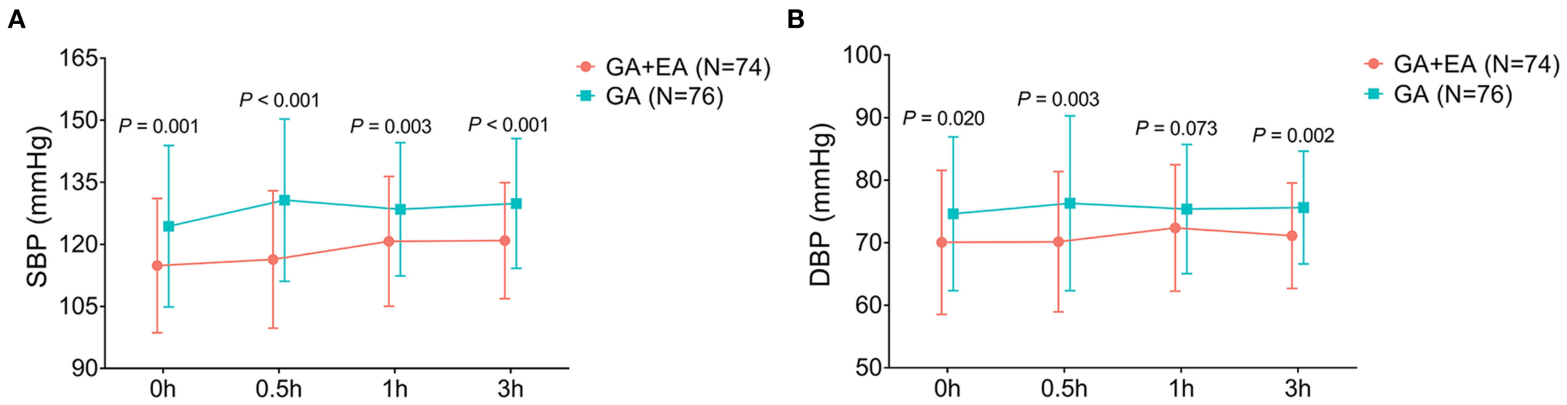
SBP and DBP. Comparison of SBP **(A)** and DBP **(B)** between GA + EA group and GA group. GA, general anesthesia; EA, epidural anesthesia; SBP, systolic blood pressure; DBP, diastolic blood pressure; h, hour.

SBP at 0 h (*P* = 0.001), 0.5 h (*P* < 0.001), 1 h (*P* = 0.003) and 3 h (*P* < 0.001) was reduced in the GA + EA group compared with the GA group (Figure 5A). Besides, DBP at 0 h (*P* = 0.020), 0.5 h (*P* = 0.003) and 3 h (*P* = 0.002) was decreased in the GA + EA group compared with the GA group, while DBP at 1 h remained similar between the two groups (*P* = 0.073) (Figure 5B).

### Comparison of HR Between Groups

**Figure 6:**
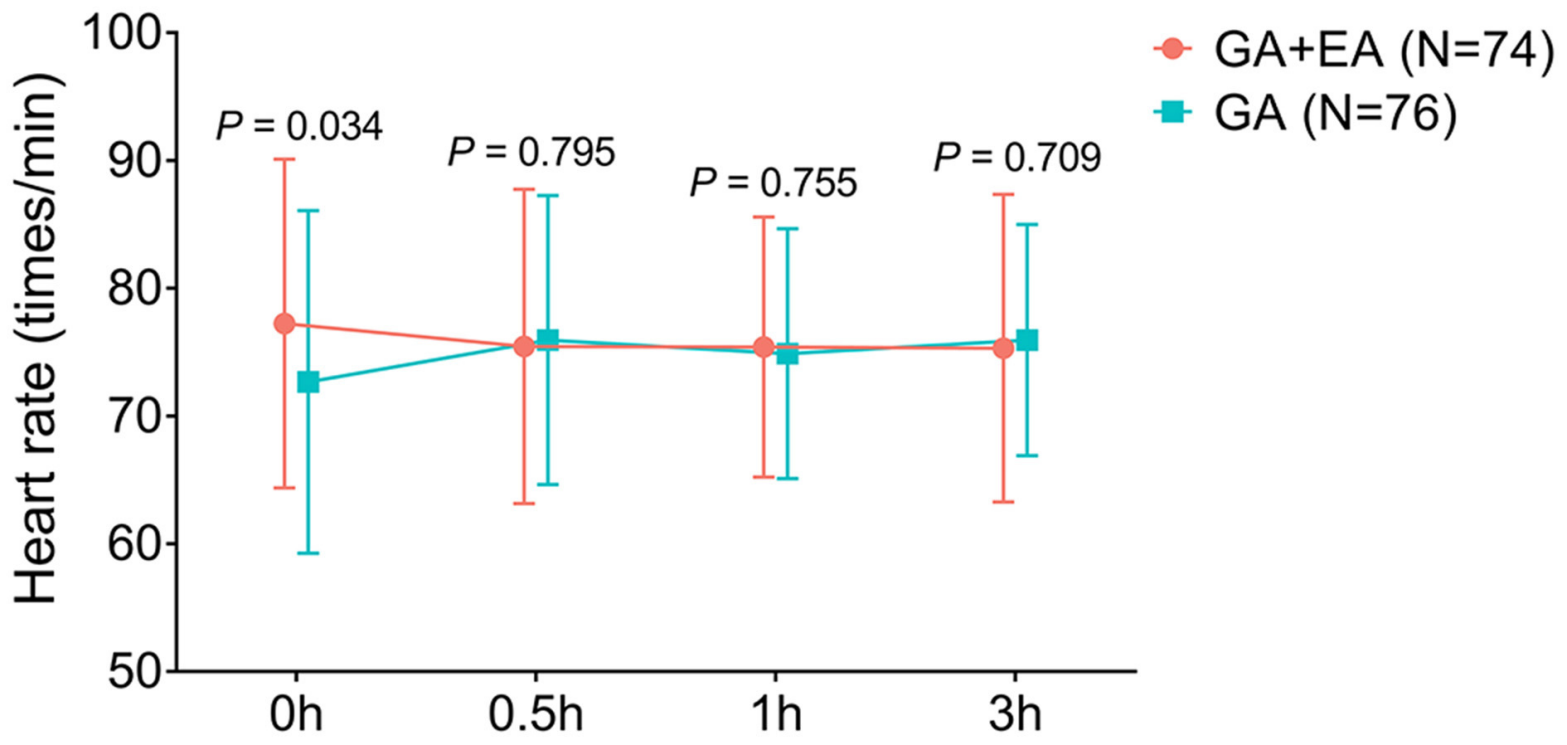
HR. Comparison of HR between GA + EA group and GA group. HR, heart rate; h, hour; GA, general anesthesia; EA, epidural anesthesia.

HR at 0 h was increased in the GA + EA group compared to the GA group (*P* = 0.034); however, HR at 0.5, 1, and 3 h did not change between the two groups (all *P* > 0.05) (Figure 6).

### Comparison of Adverse Events

The most common adverse event was pain in both GA + EA group [24 (32.4%)] and GA group [25 (32.9%)]. Besides, the occurrence of pain, severity of pain, and occurrence of vomiting were all similar between the GA + EA group and GA group (all *P* > 0.05) (Table 2).

**Table 2:** Adverse events.

**Parameters**	**GA + EA (*N* = 74)**	**GA (*N* = 76)**	***P*-value**
Pain, No. (%)	24 (32.4)	25 (32.9)	0.952
Severity of pain, No. (%)			0.867
None	50 (67.6)	51 (67.2)	
Mild	23 (31.0)	22 (28.9)	
Moderate	1 (1.4)	3 (3.9)	
Severe	0 (0.0)	0 (0.0)	
Vomiting, No. (%)	6 (8.1)	4 (5.3)	0.530

*GA, general anesthesia; EA, epidural anesthesia*.

## Discussion

CRBD commonly occurs in patients who undergo urinary catheterization, and numerous studies have explored novel potential management for CRBD (4, 5). For example, a randomized controlled trial finds that 1.5 mg/kg of tramadol reduces the occurrence and severity of CRBD compared with 1.0 mg/kg of tramadol and placebo (19). Besides, another randomized, double-blinded, controlled trial shows that intravenous administration of lidocaine decreases the occurrence of moderate-to-severe CRBD, reduces opioid requirement after the operation, and increases patients' satisfaction compared to placebo (20). However, regarding general anesthesia plus epidural anesthesia [which reduces bladder contraction and anesthetic dosage (7, 13)], no previous study has been conducted to explore its effect on CRBD. In this study, we found that: 1. General anesthesia plus epidural anesthesia reduced the occurrence and severity of CRBD compared with general anesthesia alone. These data might be explained by that: epidural anesthesia (which was performed between T11-L1 of the spinal cord) suppressed the sympathetic impulse that was related to urinary catheterization, which further impaired the detrusor activity and bladder contraction (11–13). Therefore, general anesthesia plus epidural anesthesia reduced the occurrence and severity of CRBD in patients who underwent abdominal operation with urinary catheterization. 2. Meanwhile, it was also observed that in male patients and patients older than or equal to 50 years, general anesthesia plus epidural anesthesia was more effective in reducing the occurrence and severity of CRBD, which emphasized its potential application in preventing CRBD in those patients. According to previous studies, CRBD is more likely to occur in female patients and younger patients (2, 21), which may increase the difficulty in preventing the occurrence and reducing the severity of CRBD in those patients. 3. Moreover, we also found that general anesthesia plus epidural anesthesia decreased SBP and DBP compared with general anesthesia, which could be explained by that: general anesthesia plus epidural anesthesia might have a better effect on arterial vasodilation than general anesthesia (8); 4. Besides, a slight increase of HR at 0 h was observed in patients who received general anesthesia plus epidural anesthesia compared with those who received general anesthesia only, and further studies were encouraged to verify this finding and to explore the underlying mechanisms. In addition, the patient regained consciousness and mobility 2–4 h after the epidural analgesia pump was pulled out (data not shown).

The adverse events related to epidural anesthesia in patients who receive abdominal operation have been reported by previous studies. For instance, one previous study suggests that in patients who undergo laparoscopic appendectomy, the incidences of urinary retention and postoperative nausea and vomiting are higher in patients who receive epidural anesthesia compared to those who receive general anesthesia (22). Another interesting previous study finds that in patients who undergo laparoscopic cholecystectomy, the most frequently occurred adverse events related to epidural anesthesia are urinary retention as well as nausea and vomiting (23). However, in patients who received abdominal operation with urinary catheterization, the adverse events related to general anesthesia plus epidural anesthesia were largely unclear. In the present study, we found that general anesthesia plus epidural anesthesia did not increase the occurrence of pain, severity of pain, or the occurrence of vomiting compared with general anesthesia. Apparently, urinary retention did not occur since all patients were treated with urinary catheterization. In addition, urinary retention after removal of the catheter did not occur in both groups (data not shown). Besides, different definitions of adverse events led to the differences in the occurrence of adverse events between previous studies and our present study.

Although we had found several interesting results, there were some limitations in this study. Firstly, the sample size of this study was relatively small, which may lead to low statistical power; thus, further studies with larger sample sizes could be conducted to verify the effect of general anesthesia plus epidural anesthesia on CRBD in patients who underwent abdominal operation with urinary catheterization. Secondly, the assessment of CRBD severity was based on patients' behavior, thus there might exist subjective bias. Thirdly, the parasympathetic fiber in the sacral region of the spinal cord also partly regulates bladder contraction (13); therefore, further studies could be conducted to explore the effect of epidural anesthesia (performing on S2–S4 of the spinal cord) on CRBD in patients who underwent abdominal operation with urinary catheterization. Fourthly, the effect of muscarinic receptor antagonists plus epidural anesthesia on CRBD in patients who underwent abdominal operation with urinary catheterization could be further explored. Fifthly, several potential confounding factors existed in this study, such as pelvic, vaginal, rectal surgeries and others.

## Conclusion

Collectively, general anesthesia plus epidural anesthesia reduces the occurrence and severity of CRBD with acceptable tolerance compared with general anesthesia in patients who undergo abdominal operations with urinary catheterization, indicating that general anesthesia plus epidural anesthesia may be taken into consideration for the management of abdominal operation-related CRBD.

## Data Availability Statement

The original contributions presented in the study are included in the article/Supplementary Material, further inquiries can be directed to the corresponding author/s.

## Ethics Statement

The studies involving human participants were reviewed and approved by the Institutional Review Board of Ruijin Hospital, Shanghai Jiaotong University School of Medicine and Fudan University Shanghai Cancer Center. The patients/participants provided their written informed consent to participate in this study.

## Author Contributions

SS and CW made substantial contributions to the design of the present study. Data acquisition and interpretation was performed by SS, CW, JZ, and PS. JZ and PS critically revised the manuscript for important intellectual content. All author approved the final version of the manuscript. All authors agree to be accountable for all aspects of the work in ensuring that questions related to the accuracy or integrity of the work are appropriately investigated and resolved.

## Conflict of Interest

The authors declare that the research was conducted in the absence of any commercial or financial relationships that could be construed as a potential conflict of interest.

## Publisher's Note

All claims expressed in this article are solely those of the authors and do not necessarily represent those of their affiliated organizations, or those of the publisher, the editors and the reviewers. Any product that may be evaluated in this article, or claim that may be made by its manufacturer, is not guaranteed or endorsed by the publisher.
