# Supplementary material for: Occurrence and Severity of Catheter-Related Bladder Discomfort of General Anesthesia Plus Epidural Anesthesia vs. General Anesthesia in Abdominal Operation With Urinary Catheterization: A Randomized, Controlled Study
Source: Front Surg. 2021 Sep 6;8:658598. doi: 10.3389/fsurg.2021.658598 (PMC8450512; doi:10.3389/fsurg.2021.658598)
Supplement: Supplementary file 1 [file Table_1.DOCX]

**Supplementary table 1.** Operations of patients.

| Items | GA+EA (N=74) | GA (N=76) |
| --- | --- | --- |
| Adnexectomy | 2 (2.7) | 5 (6.6) |
| Caudal pancreatectomy | 3 (4.1) | 2 (2.6) |
| Cystectomy | 1 (1.4) | 0 (0.0) |
| Exploratory laparotomy | 1 (1.4) | 1 (1.3) |
| Gastrectomy | 10 (13.5) | 1 (1.3) |
| Hemicolectomy | 3 (4.1) | 3 (3.9) |
| Hemihepatectomy | 0 (0.0) | 1 (1.3) |
| Hepatectomy | 0 (0.0) | 1 (1.3) |
| Hepatic lobectomy | 1 (1.4) | 0 (0.0) |
| Hysterectomy | 17 (23.0) | 12 (15.8) |
| Intestine neostomy | 0 (0.0) | 4 (5.3) |
| Laparoscopic colectomy | 0 (0.0) | 1 (1.3) |
| Laparoscopic exploratory laparotomy | 0 (0.0) | 1 (1.3) |
| Laparoscopic fenestration for liver cyst | 0 (0.0) | 1 (1.3) |
| Laparoscopic gastrectomy | 2 (2.7) | 0 (0.0) |
| Laparoscopic hemicolectomy | 0 (0.0) | 1 (1.3) |
| Laparoscopic hepatoma resection | 1 (1.4) | 1 (1.3) |
| Laparoscopic LAR | 2 (2.7) | 1 (1.3) |
| Laparoscopic myomectomy | 0 (0.0) | 1 (1.3) |
| Laparoscopic nephrectomy | 4 (5.4) | 3 (3.9) |
| Laparoscopic pancreatic resection | 2 (2.7) | 4 (5.3) |
| Laparoscopic proctectomy | 0 (0.0) | 1 (1.3) |
| Laparoscopic prostatectomy | 2 (2.7) | 6 (7.9) |
| Laparoscopic surgery for renal cyst | 1 (1.4) | 0 (0.0) |
| LAR (Dixon) | 1 (1.4) | 1 (1.3) |
| Miles surgery | 3 (4.1) | 0 (0.0) |
| Nephrectomy | 1 (1.4) | 1 (1.3) |
| Ovarian cyst dissection | 0 (0.0) | 1 (1.3) |
| Pancreatectomy | 3 (4.1) | 0 (0.0) |
| pancreaticoduodenectomy | 2 (2.7) | 0 (0.0) |
| Panhysterectomy | 3 (4.1) | 12 (15.8) |
| Partial nephrectomy | 0 (0.0) | 2 (2.6) |
| Pelvic lymph node dissection | 0 (0.0) | 1 (1.3) |
| Pelvis resection | 0 (0.0) | 2 (2.6) |
| Peritoneal tumor resection | 1 (1.4) | 0 (0.0) |
| Retroperitoneal pelvis resection | 0 (0.0) | 1 (1.3) |
| Retroperitoneal TRS | 5 (6.8) | 3 (3.9) |
| Sigmoidectomy | 3 (4.1) | 1 (1.3) |

GA, general anesthesia; EA, epidural anesthesia; TRS, tumor reductive surgery; LAR, low anterior rectal resection
