# Supplementary material for: Occurrence and Severity of Catheter-Related Bladder Discomfort of General Anesthesia Plus Epidural Anesthesia vs. General Anesthesia in Abdominal Operation With Urinary Catheterization: A Randomized, Controlled Study
Source: Front Surg. 2021 Sep 6;8:658598. doi: 10.3389/fsurg.2021.658598 (PMC8450512; doi:10.3389/fsurg.2021.658598)
Supplement: Supplementary file 2 [file Table_2.DOCX]

**Supplementary table 2.** Operative regions or organs of patients.

| Items | GA+EA (N=74) | GA (N=76) |
| --- | --- | --- |
| Uterus | 20 (27.0) | 25 (32.9) |
| Urinary bladder | 1 (1.4) | 0 (0.0) |
| Stomach | 12 (16.2) | 1 (1.3) |
| Small intestine | 0 (0.0) | 4 (5.3) |
| Rectum | 11 (14.9) | 6 (7.9) |
| Prostate | 2 (2.7) | 6 (7.9) |
| Peritoneum | 1 (1.4) | 0 (0.0) |
| Pelvis | 0 (0.0) | 4 (5.3) |
| Pancreaticoduodenum | 2 (2.7) | 0 (0.0) |
| Pancreas | 8 (10.8) | 6 (7.9) |
| Ovarian | 0 (0.0) | 1 (1.3) |
| Liver | 2 (2.7) | 4 (5.3) |
| Kidney | 6 (8.1) | 6 (7.9) |
| Colon | 6 (8.1) | 6 (7.9) |
| Adnexa uteri | 2 (2.7) | 5 (6.6) |
| Abdomen | 1 (1.4) | 2 (2.6) |

GA, general anesthesia; EA, epidural anesthesia.
